# CD47-Independent Effects Mediated by the TSP-Derived 4N1K Peptide

**DOI:** 10.1371/journal.pone.0098358

**Published:** 2014-05-21

**Authors:** Pascal Leclair, Chinten James Lim

**Affiliations:** 1 Department of Pediatrics, University of British Columbia, Vancouver, British Columbia, Canada; 2 Department of Cell and Developmental Biology, University of British Columbia, Vancouver, British Columbia, Canada; 3 Child and Family Research Institute, BC Children's Hospital, Vancouver, British Columbia, Canada; University of Toledo, United States of America

## Abstract

4N1K is a peptide fragment derived from the C-terminal, globular domain of thrombospondin which has been shown to mediate integrin-dependent cell adhesion and promote integrin activation acting via the cell-surface receptor, CD47. However, some studies found that 4N1K could act independently of CD47, putting in question the specificity of 4N1K for CD47. This led us to characterize the cellular and non-cellular effects of 4N1K. We found that 4N1K stimulated a potent increase in binding of a variety of non-specific IgG antibodies to cells in suspension. We also found that these same antibodies, as well as CD47-deficient cells, could bind substrate-immobilized 4N1K significantly better than a control peptide, 4NGG. Furthermore, we found that cells treated with 4N1K at higher concentrations inhibited, while lower concentrations promoted cell adhesion to immobilized fibronectin as an integrin substrate. Importantly, both the stimulatory and the inhibitory activity of 4N1K occurred as efficiently in the CD47-deficient JinB8 cells, as it did in the CD47-expressing parental or in JinB8 cells reconstituted with CD47 expression. Given these results, we suggest that 4N1K interacts non-specifically with epitopes commonly found on the cell surface, and conclude that it is not a suitable peptide for use to study the consequences of CD47 receptor ligation.

## Introduction

Integrins are a family of cell adhesion receptors that can be regulated by conformational changes in their extracellular domains which modulate their affinity state for binding to ligands [1]. Regulation of integrin activation is a key step in cellular adhesive functions and is required for efficient arrest of cells during recruitment to sites of inflammation [2], [3]. CD47, also known as “Integrin Associated Protein” (IAP) is a penta-spanning receptor protein with a highly glycosylated, IgV-containing extracellular domain that mediates binding to signal regulatory protein (SIRP) and to thrombospondins (TSP) [4]–[6]. CD47 has been shown to regulate cell adhesion and spreading, and was found to act as a “don't eat me” signal since cells with significant levels of CD47 expression exhibit reduced potential for engulfment by professional phagocytes [7], [8].

Thrombospondins are a multimeric, multidomain, and multifunctional family of proteins that are secreted by a variety of cells during normal and pathological conditions, and can subsequently be incorporated into the extracellular matrix [9]. All five members of the TSP family share a common C-terminal domain containing a VVM motif that mediates cell binding to CD47 [4], [9]. The 10-mer KRFYVVMWKK peptide, commonly known as 4N1K, is derived from the C-terminal globular domain that was characterized as the main site responsible for TSP-mediated cell adhesion [10], [11]. CD47 was subsequently determined to be the receptor responsible for 4N1K-mediated cell adhesion since adhesion to this peptide was enhanced in CD47-transfected OV10 cells compared to non-transfected cells [12].

4N1K has been shown to regulate integrin-mediated adhesive functions in several cell systems, including observations whereby 4N1K was found to decrease cell adhesion to immobilized TSP [13]–[15], to endothelium monolayers [16], and to integrin ligand substrates such as laminin [10], collagen [17] and fibronectin [18]. However, other reports have shown that 4N1K could increase adhesion to some of the same ligands [14], [19], [20]. Furthermore, 4N1K was found to promote integrin activation in several reports where Ligand-Induced Binding Site (LIBS) antibodies were used as reporters to assess the high-affinity state of integrins [21]. Still, other reports using CD47-deficient cell lines have found that 4N1K could mediate effects that is independent of CD47 receptor expression [8]. For example, 4N1K-mediated aggregation of platelets was found to be similar in wild type and CD47-deficient murine cells [22], whereas another study found that soluble 4N1K induced adhesion of CD47^+/+^ cells as efficiently as CD47^−/−^ cells [19].

Two of the three SIRP isoforms, SIRPα and SIRPγ, have been characterized as ligands for CD47 [23], [24]. In particular, a disulfide bridge between the extracellular and membrane spanning domains of CD47 was found to be critical for CD47 binding to SIRPα [25]. The solved crystal structure of the CD47-SIRPα complex revealed that the interaction occurs via a four-loop structure in the IgV domain of SIRPα and a two-loop structure in the IgV domain of CD47 [26]. In contrast, plasmon resonance studies that confirmed the CD47-SIRPα interaction were unable to detect an interaction between CD47 and a TSP1-fragment termed the signature domain comprising the C-terminal domain downstream of the last three type3 repeats [27]. Furthermore, crystal structures of the TSP1 and TSP2 signature domains revealed that the globular domain of TSP is composed of several β-strands arranged in a jelly roll formation homologous to L-lectin type domains [28], [29]. This β-strand arrangement contains the VVM motif of 4N1K, which was found to be buried within a cleft formed by two hydrophobic domains, making accessibility of this sequence to CD47 unlikely without significant conformational changes in the globular domain of TSP.

The purpose of this study, given the discrepancies ascribed to the effects of CD47 ligation by 4N1K, was to characterize the non-specific effects of 4N1K on integrin functions. What we found was that a number of reported activities attributed to 4N1K acting as a ligand agonist for CD47 may be explained by a phenomenon involving non-specific interactions between 4N1K and cell surface proteins in a manner independent of CD47. Via a series of carefully controlled studies, we show that Jurkat T-cells incubated with 4N1K not only exhibited increased binding to a β1-integrin LIBS antibody, but also to various other IgG antibodies in a non-specific manner. We also found that CD47-deficient Jurkat cells could bind to substrate-immobilized 4N1K just as well when compared to cells that re-express CD47. Furthermore, some of these findings can be replicated using a cell-free, antibody only assay system, leading to our suggestion that 4N1K may interact with domains commonly found in IgG antibodies. Finally, we determined that pre-incubation of cells with soluble 4N1K could block adhesion to substrate-immobilized integrin ligands in a CD47-independent manner. Therefore, the use of 4N1K to study CD47-mediated effects could result in erroneous conclusions about the effects of TSP's C-terminal binding domain and, consequently, of CD47's role in cell adhesion and integrin activation.

## Materials and Methods

### Cells and reagents

Jurkat T cells were purchased from the American Type Culture Collection (ATCC). JinB8, a CD47-deficient Jurkat derivative cell line as described in [30], was a gift from Dr. Roberts. The Jurkat 6A (parental) and the β1-deficient derivative termed A1 were provided by Dr. Shimizu, as described in [31]. All cells were maintained at 37°C, 5% CO_2_, in complete RPMI (RPMI 1640 supplemented with 10% fetal bovine serum, non-essential amino acids (NEAA, Invitrogen), and penicillin-streptomycin (Gibco)). The synthetic thrombospondin C-terminal peptide, 4N1K (KRFYVVMWKK), and its negative control peptide, 4NGG (KRFYGGMWKK), were obtained from GL Biochem. The lyophilized peptides were freshly dissolved in blank RPMI (serum-free) as a 0.525 mM stock solution and used for assays within 24 hrs of solubilization. All other reagents were purchased from Sigma unless otherwise stated. Fibronectin from human plasma was purified by affinity chromatography through gelatin-sepharose (GE Healthcare).

### Plasmids and transfections

The pKS336-hCD47 (isoform 4) expression construct was a gift from Dr.Ohdan [32]. To obtain hCD47 isoform 2, the dominant form expressed in hematopoietic cells [30], pcDNA3.1-CD47 (isoform 2) was subcloned from isoform 4 by polymerase chain reaction using the following primers: Fwd 5′-TCATTGTCATTGTTGGAGCC-3′; Rev 5′-GGATCCTCAGTTATTCCTAGGAGGTTGTATAGTC-3′. Cell transfection was performed using the Amaxa nucleofection kit V (Lonza) and CD47-expressing cells were serially sorted by flow cytometry, followed by limiting serial dilution to obtain clonal populations of CD47-expressing cells.

### Antibodies

Total β1-integrin (TS2/16) and CD47 (BRIC-126) antibodies were from Santa Cruz Biotechnology; α5-integrin (NKI-SAM-1) and mouse normal control IgG antibodies were from BioLegend; the activation-dependent β1-antibody (HUTS-21) was from BD Pharmingen. DyLight 488-conjugated goat anti-mouse (GAM) secondary antibody was from Thermo Scientific.

### Flow cytometry and integrin activation assays

All flow cytometry assays were performed at the Child and Family Research Institute Flow Core facility. Sample data acquisition was conducted on the FacsCanto instrument while fluorescence-activated cell sorting was performed on the FacsAria (BD Biosciences). Post-acquisition analysis was conducted using FlowJo (Tree Star). For integrin activation assays, 6×10^5^ cells per sample were routinely harvested and washed in room temperature (r.t.) Dulbecco's phosphate buffered saline (PBS), pH 7.4, and incubated with 500 ng HUTS-21, TS2/16, or IgG control antibody diluted in blank RPMI with 1% bovine serum albumin (BSA), in addition to a no primary control, along with indicated treatments for 30 min at 37°C. Cells were then washed in cold PBS and incubated for 20 min with 400 ng DyLight 488-conjugated secondary antibodies diluted in 1% BSA/PBS at 4°C. After a final wash in cold PBS, cells were analyzed by flow cytometry as indicated above using an excitation wavelength of 488 nm and a 530/30 emission filter.

### Protein quantification assay for adsorbed peptides

Titrating concentrations of 4N1K and 4NGG peptides were adsorbed onto wells of a 96-well plate for 1 hr at r.t. After washing the wells 2x with r.t. PBS to remove unbound peptides, a CBQCA (Invitrogen) protein quantification assay was performed according to manufacturer's instructions to quantify the amount of peptide adsorbed in each well.

### Peptide-antibody interaction assay

50 µM 4N1K, 50 µM 4NGG, 50 µM Poly-L-Lysine (PLL), or 1% BSA/PBS, were adsorbed onto wells of a 96-well plate for 1 hr at r.t. and rinsed 3x in r.t PBS. 50 µL of primary antibodies diluted to 2.5 ng/µL in PBS were then added and incubated for 20 min at 4°C before rinsing 2x in cold PBS, followed by incubation with 50 µL Dylight 488-conjugated secondary antibodies diluted to 4 ng/µL in PBS at 4°C for 20 min. After a final wash in cold PBS, fluorescence was assessed using an Enspire spectrophotometer (Perkin Elmer) at 485 nm excitation and 515 nm emission. For peptide titration assays, the indicated peptide concentrations refer to the coating solution.

### Cell adhesion assays

Wells of a 96-well dish were substrate-coated and washed 3x in PBS prior to seeding with cells. Plasma fibronectin coating was performed at 20 µg/mL dissolved in PBS. Peptide coating is as described in previous paragraph. Prior to plating, cells were labeled with CellTracker Green CMFDA (Invitrogen) according to manufacturer's instructions. 2×10^5^ cells were plated in each well and incubated at 37°C for 20 mins for adhesion to peptides and 45 min for adhesion to fibronectin. Fluorescence readings were acquired with an Enspire spectrophotometer (485 nm excitation, 515 nm emission) both before and following 2x washes with PBS. Background fluorescence from the incubation medium was subtracted from each reading and % adhesion was calculated as follows: (Fluorescence after washes/Initial fluorescence)_*_100.

### Statistical analysis

The Student's unpaired *t* test was used for all statistical analyses in this report.

## Results

**Figure 1:**
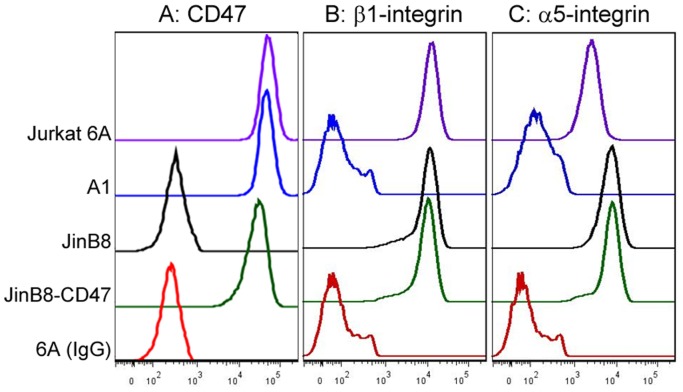
Expression of CD47, β1-integrin and α5-integrin receptors on various Jurkat cell lines used. 6A is the wildtype Jurkat T-cells and parental strain to A1, which is deficient in β1-integrin expression. JinB8 is a CD47-deficient derivative of Jurkat cells, while JinB8-CD47 is stably reconstituted with expression of CD47 isoform 2. Cells were harvested, washed with PBS and incubated with the indicated primary antibodies. Following washes and incubation with fluorophore-conjugated secondary antibodies, bound antibodies was assessed by flow cytometry as an indicator of receptor expression levels. Normal mouse IgG was used as a non-specific primary antibody control.

**Figure 2:**
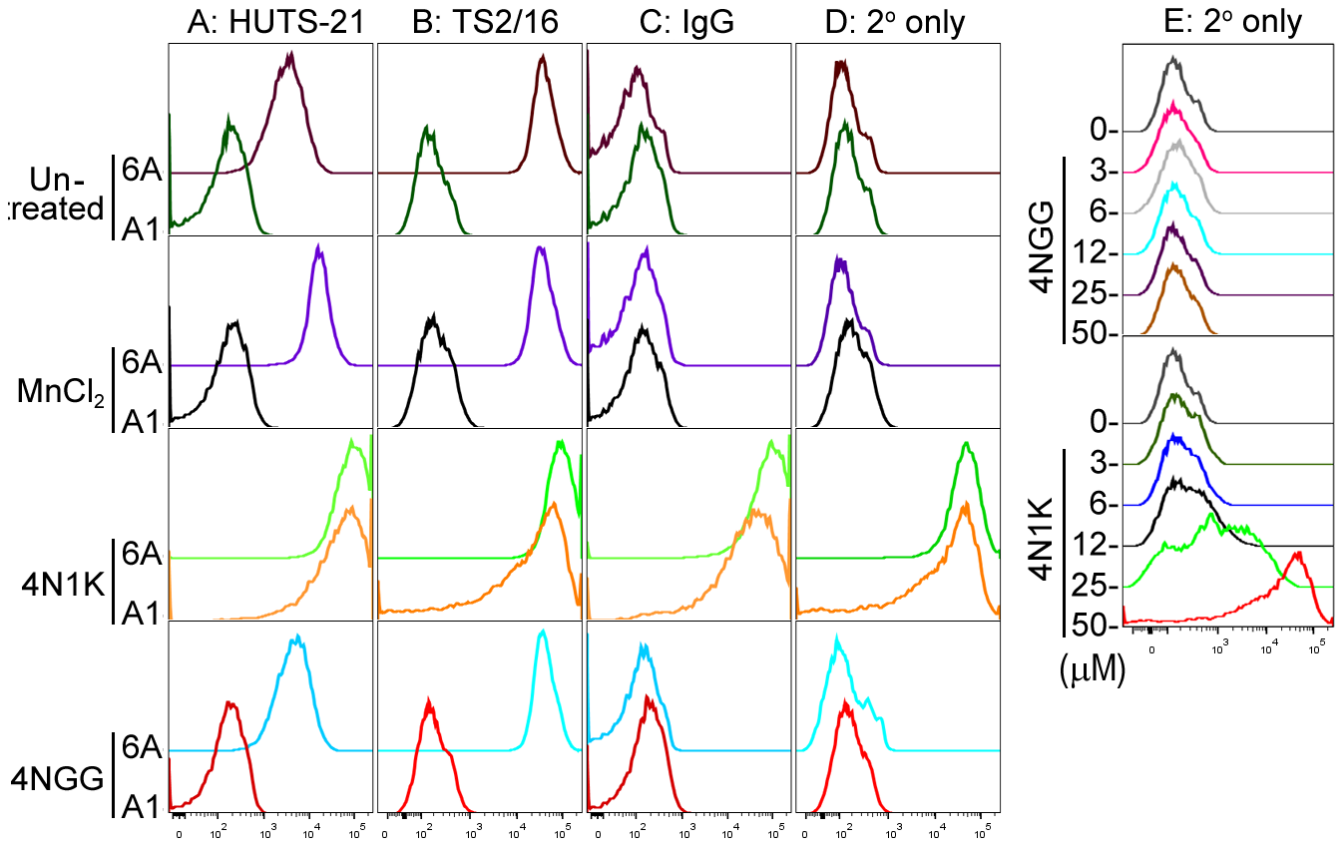
Incubation of Jurkat cells with 4N1K results in non-specific binding of various antibodies. Jurkat 6A or A1 cells were incubated without or with treatment (1 mM MnCl_2_, 50 µM 4N1K or 50 µM 4NGG) for 30 min at 37°C, along with one of three antibodies: A) HUTS-21 to detect activated epitopes of β1-integrins, B) TS2/16 to detect total β1-integrins, C) a non-specific IgG control antibody, and D) no primary antibody control treatment. Cells were then washed, incubated with fluorophore-conjugated secondary antibody, and antibody binding was assessed using flow cytometry. E) Flow cytometry data of A1 cells incubated with the indicated concentrations of 4NGG or 4N1K peptide and fluorophore-conjugated secondary antibody. Histogram data as shown is for one experiment that is representative of three independently conducted experiments.

Previous reports that have made use of 4N1K to study the ligation of CD47 on integrin functions yielded seemingly contradictory results that were either stimulatory or inhibitory [20]–[22], [33]. In an effort to clarify this, we first assembled a panel of Jurkat (6A) and Jurkat-derivative cell lines that include A1 (integrin β1-deficient), JinB8 (CD47-deficient) and JinB8-CD47 (reconstituted expression with CD47 isoform 2). The corresponding CD47, integrin β1, and integrin α5 receptor expression levels were assessed by flow cytometry, as shown in Fig. 1. To assess the effect of soluble 4N1K peptide on cellular β1-integrin activation, we used the β1-specific and activation-dependent LIBS antibody, HUTS-21 [34]. Here, we used MnCl_2_ as a positive control for integrin activation since this reagent has been shown to induce the high affinity conformation of integrins to which HUTS-21 binds [34]. As expected, incubation of wild-type Jurkat 6A cells with MnCl_2_ resulted in increased HUTS-21 antibody binding when compared to the untreated controls, indicating Mn^2+^ -mediated stimulation of β1-integrin activation (Fig. 2A). As a negative control for HUTS-21 binding, we used the β1-deficient A1 cells. As expected, A1 cells exhibited only minimal binding to HUTS-21 in a manner that was not changed with MnCl_2_ treatment (Fig. 2A). Compared to untreated or 4NGG-treated controls, MnCl_2_ treatment did not alter binding of 6A or A1 cells to either: an activation-independent β1-integrin antibody TS2/16 (Fig. 2B), a non-specific mouse IgG control antibody (Fig. 2C), or to the goat anti-mouse (GAM) secondary antibody used for all assays (Fig. 2D). Unexpectedly, incubation of 6A or A1 cells with 4N1K induced a dramatic increase in HUTS-21 antibody binding to a level that is greater than that seen with MnCl_2_ (Fig. 2A). Furthermore, 4N1K-treated cells exhibited greatly increased binding of TS2/16, a non-specific mouse IgG control, or of a GAM secondary antibody only control (Fig. 2B–D). Since binding of the fluorophore-conjugated secondary antibody alone is sufficient to explain the ‘pseudo’ integrin activation data, we titrated the concentration of 4N1K incubated with the cells to show that GAM secondary antibody binds to A1 cells in a dose-dependent manner (Fig. 1E). This effect was not observed for untreated or control 4NGG peptide-treated cells, suggesting the phenomenon observed as a property unique to the 4N1K peptide. Importantly, our assay shows that cell incubation with soluble 4N1K peptide resulted in non-specific binding of several IgG antibodies to the cell surface in a manner independent of integrins. Thus, it is impossible to infer the effects of 4N1K on cellular integrin activation when a LIBS antibody-based assay is used.

**Figure 3:**
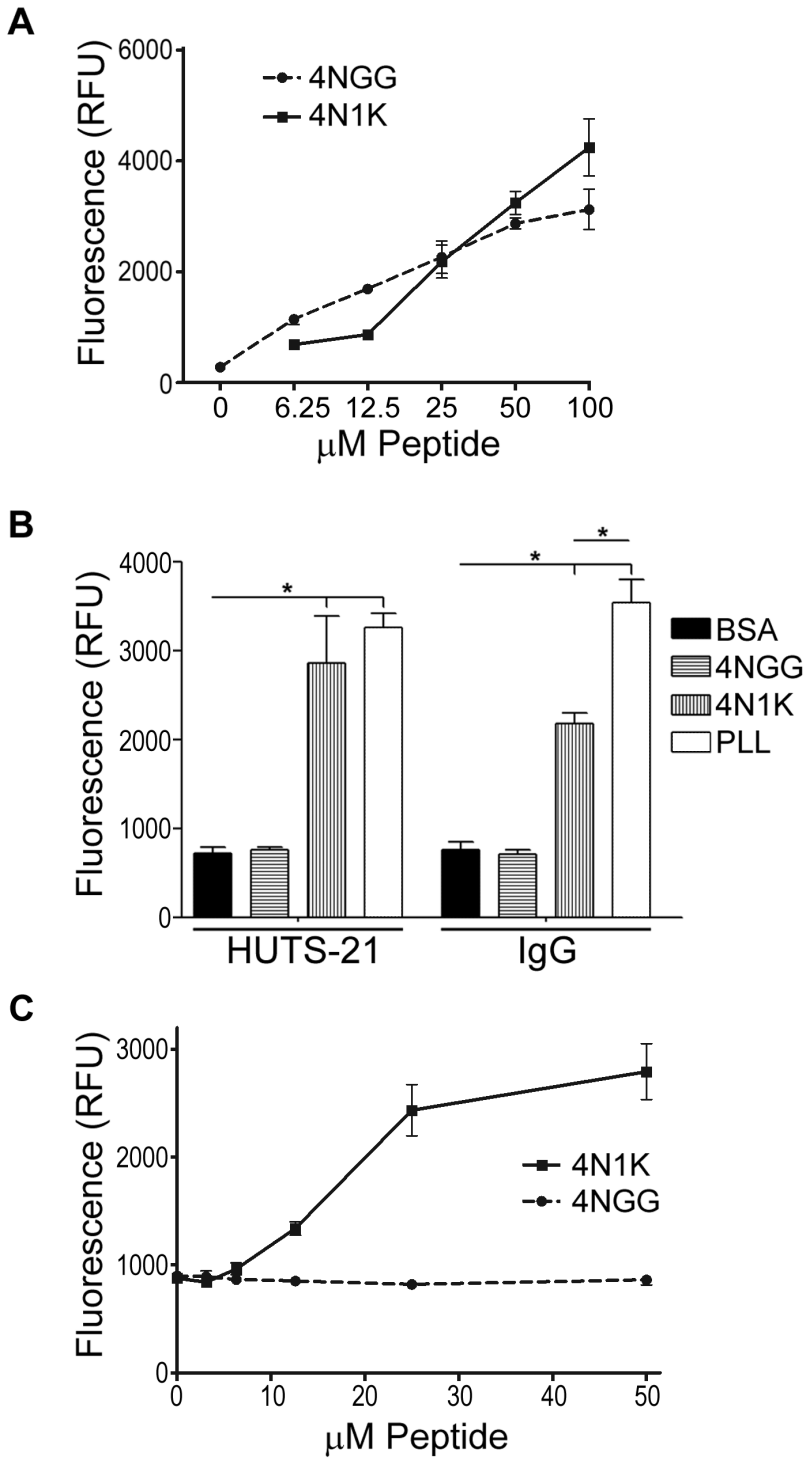
Immobilized 4N1K binds non-specifically to several IgG antibodies. A) Wells of a 96-well dish were coated with the indicated concentrations of peptides and washed with PBS. The amount of adsorbed peptides was quantified using the CBQCA protein assay kit as indicated in methods and materials. The plotted data represent the means ± standard deviation of three replicate wells of one experiment that is representative of three independently conducted ones. B) HUTS-21 or an IgG isotype control antibody were added to wells pre-coated with 50 µM 4NGG, 50 µM 4N1K, 50 µM poly-L-lysine, or 1%BSA/PBS. Following washes and incubation with a secondary antibody, bound antibodies were detected using a microplate spectrofluorometer. Data as plotted are the means ± standard deviation for three replicate samples of one experiment (*, p<0.05) that is representative of three independently conducted experiments. C) Same as in B) but titrating amounts of 4NGG and 4N1K were adsorbed to wells and only secondary antibodies were added.

The cell-based assays conducted thus far suggested interactions mediated by 4N1K with IgG-containing proteins. These observations prompted us to test the ability of 4N1K to interact with antibodies that had not been raised against 4N1K (or TSP) in an ELISA-type, cell-free system. We first performed a protein quantification assay to show that both 4N1K and 4NGG peptides efficiently adsorbed to the plastic wells of a 96-well dish in a concentration-dependent manner (Fig. 3A). Then, we adsorbed 4N1K, 4NGG, BSA, or the highly adhesive cationic polypeptide poly-L-lysine (PLL), onto wells of a 96-well dish, followed by addition of primary and/or secondary antibodies as described in methods and materials. As shown in Fig. 3B, both 4N1K or PLL, but not 4NGG nor BSA, significantly bound HUTS-21 and normal mouse IgG control antibodies. Since co-incubation with 4N1K promoted binding of GAM secondary antibodies to cells (Fig. 2E), we assessed the ability of the adsorbed peptides to bind the fluorophore-conjugated GAM secondary antibodies. As shown in Fig. 3C, adsorbed 4N1K, but not 4NGG, bound GAM secondary antibodies in a manner dependent on the peptide coating concentration. To ensure that this was not an IgG species, isotype, or fluorophore-specific phenomenon, we repeated the assay using a variety of secondary antibodies (goat anti-rat FITC, donkey anti-goat DyLight800, and goat anti-rabbit DyLight633) and obtained similar results (not shown). These data indicate that immobilized 4N1K interacts with a variety of IgG proteins in a promiscuous manner.

**Figure 4:**
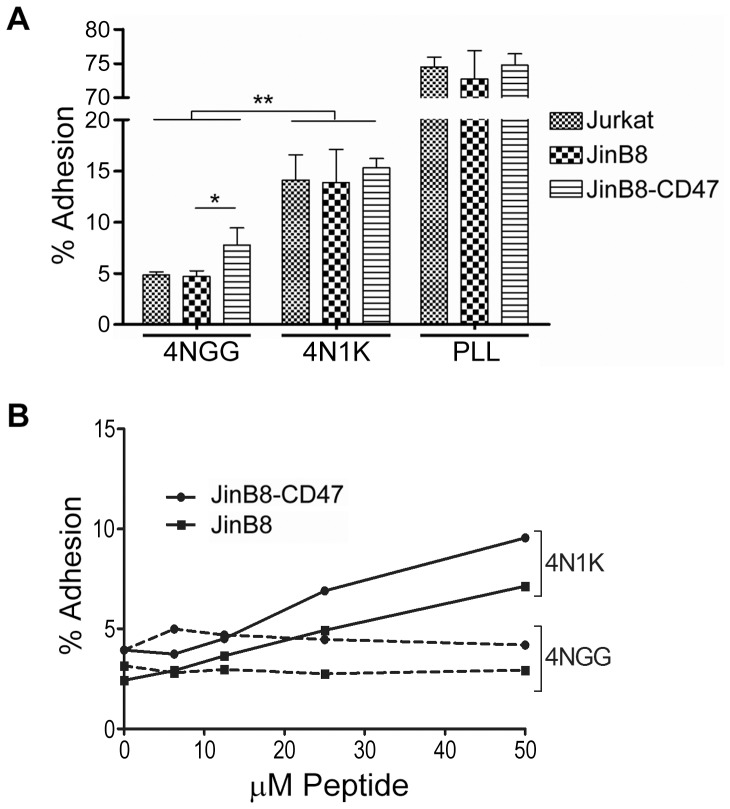
Cells bind to immobilized 4N1K independently of CD47 expression. A) Jurkat, JinB8, or JinB8-CD47 cells pre-labeled with Celltracker Green were added to wells pre-coated with 50 µM 4NGG, 50 µM 4N1K, or 50 µM poly-L-lysine, and incubated for 20 min. In-well fluorescence was measured using a spectrofluorometer both before and after washes to remove non-adherent cells, and % adhesion was calculated as stated in methods and materials. Data as plotted are the means ± standard deviation for three replicate samples of one experiment (*, p<0.05; **, p<0.002) that is representative of three independently conducted experiments. B) Same as in A) but with titrating amounts of 4NGG and 4N1K pre-adsorbed to wells. Data as plotted are the means for samples conducted in duplicates.

The fact that no cellular membrane proteins were involved in the previous assay (Fig. 3) led us to speculate on the nature of 4N1K's interactions with the cell. The immunoglobulin-like extracellular domain of CD47 mediates binding to ligands [5], [27]. Previous studies have shown that immobilized 4N1K can act as an adhesion substrate presumed to engage the CD47 receptor [12], [35]. To assess the requirement of CD47 in cellular adhesive interactions with immobilized substrates, we compared the ability of Jurkat, the CD47-deficient derivative cell line JinB8 [30], and the CD47-reconstituted JinB8-CD47 cells to engage 4N1K-, 4NGG-, or PLL-coated dishes. We found that all three cell lines bound immobilized 4N1K to a similar extent, which was significantly greater than 4NGG but at much reduced efficiency than for PLL (Fig. 4A). Furthermore, JinB8 and JinB8-CD47 cells bound immobilized 4N1K in a manner dependent on the concentration of the coating peptide (Fig. 4B). We noted that JinB8-CD47 cells were consistently more adhesive when compared to JinB8 cells, as evidenced by increased adhesion on uncoated or 4NGG-coated wells (Fig. 4A,B). Importantly, our data shows that the absence of CD47 bore little consequence on the cellular interaction with immobilized 4N1K, raising the possibility that 4N1K simply enabled the capture of cells via CD47-independent and non-specific surface protein interactions.

**Figure 5:**
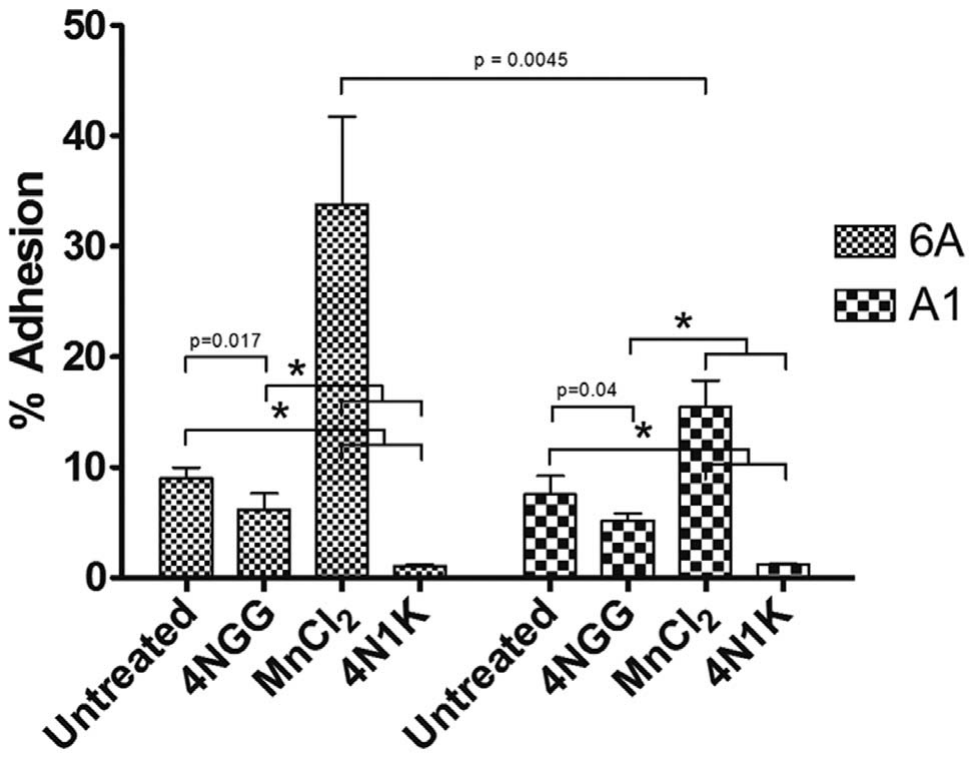
Cell adhesion to fibronectin is decreased with 50 µM 4N1K treatment. Jurkat 6A and A1 cells were labeled with Celltracker Green, then treated with buffer, 50 µM 4NGG, 50 µM 4N1K, or 1 mM MnCl_2_ for 20 min prior to seeding onto fibronectin-coated wells. In-well fluorescence was measured using a spectrofluorometer both before and after washes to remove non-adherent cells, and % adhesion was calculated as stated in methods and materials. Data as plotted are the means ± standard deviation for three replicate samples of one experiment (*, p<0.001).

Previous studies have shown that incubation of cells with 50 µM or higher soluble 4N1K could block integrin-dependent cell adhesion [10], [16], [18]. To determine if we could reproduce this phenomenon, cells were left untreated or treated with soluble 4N1K or 4NGG peptides prior to seeding on fibronectin-coated dishes to assess integrin-mediated adhesion. Consistent with previous studies using integrin ligand-coated dishes as a substrate [17], [18], pre-incubation with 4N1K resulted in a significant decrease in adhesion for both Jurkat 6A and A1 (β1-deficient) cells when compared to untreated or 4NGG-treated conditions (Fig. 5). As a positive control for stimulation of integrin-mediated adhesion, we show that cells treated with MnCl_2_ exhibit increased adhesion to fibronectin (Fig. 5). This increase was less prominent in A1 cells, presumably due to the deficiency in β1-integrin expression (Fig. 1).

**Figure 6:**
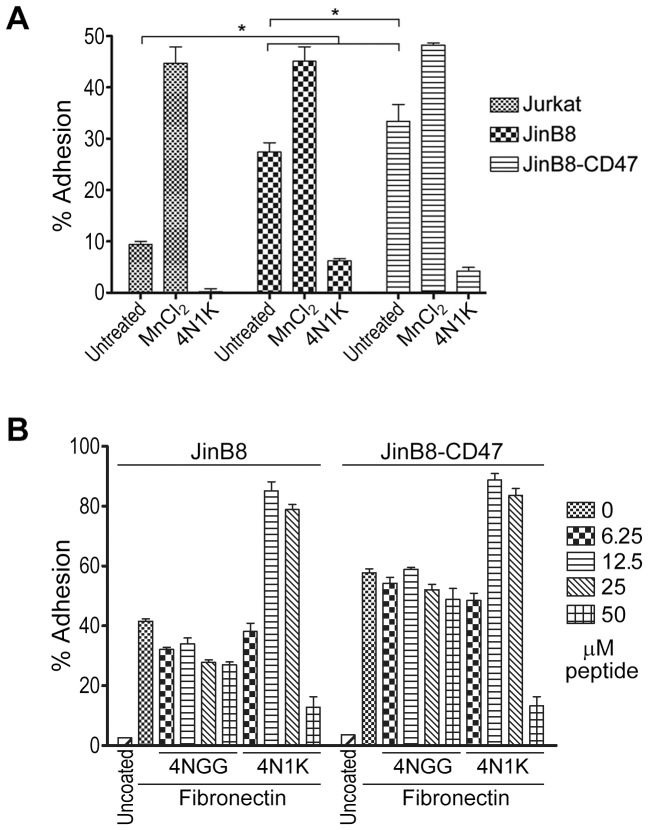
4N1K-mediated inhibition of adhesion occurs in a CD47-independent manner. A) Jurkat, JinB8 and JinB8-CD47 cells were labeled with Celltracker Green, then treated with buffer, 50 µM 4N1K, or 1 mM MnCl_2_ for 20 min prior to seeding onto fibronectin-coated wells and % adhesion calculated as stated before. Data as plotted are the means ± standard deviation of three replicate samples of one experiment (*, p<0.05) that is representative of three independently conducted ones. B) Same as in A) but with titrating amounts of 4NGG and 4N1K treatment of cells.

To determine if the 4N1K-mediated block in cell adhesion was mediated by CD47, we repeated the assay with CD47-deficient and CD47-expressing cells. As shown in Fig. 6A, pre-treatment with 50 µM 4N1K blocked adhesion of Jurkat, JinB8, and JinB8-CD47 cells, again pointing to a CD47-independent mechanism of action by 4N1K. We also titrated the concentration of co-incubated peptides to determine if a lower concentration may achieve CD47-specific effects. The results (Fig. 6B) were somewhat unexpected as we found that 4N1K induced both JinB8 and JinB8-CD47 cell adhesion to fibronectin at lower peptide concentrations (12.5 to 25 µM). Thus, soluble 4N1K had opposing effects on cell adhesion to integrin substrates depending on its concentration, although these effects were not significantly modified by the absence of CD47, its presumed receptor.

We also noted that the JinB8 derivative cell line, even in the absence of treatment, demonstrated higher levels of adhesion to fibronectin when compared to their parental Jurkat cells (Fig. 6A). To gain an understanding for this observation, we compared the expression of integrins α5 and β1, as well as CD47. While β1-integrins were comparable for all cell lines assessed, we observed that both JinB8 and JinB8-CD47 have increased α5 expression when compared to Jurkat cells (Fig. 1). While fibronectin is a rather non-specific substrate able to engage multiple integrins, our results are consistent with the fact that fibronectin is the preferred substrate for α5β1 integrins [36]. Due to the inherent difference in receptor expression between these cell lines, we urge caution in using Jurkat as the parental control for JinB8 cells, particularly when integrin-mediated functions are assayed. We did find that reconstituted CD47 expression in JinB8 cells led to a significant increase in adhesion to fibronectin under basal (untreated) conditions (Fig. 6A,B), in agreement with a positive stimulatory role of CD47 on integrin function [37].

## Discussion

The 4N1K peptide that is derived from the C-terminus of thrombospondin has been used numerous times over the years as a ligand to study CD47, a cell-surface receptor with an extracellular IgV domain that has been shown to regulate integrin function [4]. Here, we first used HUTS-21, a LIBS antibody that specifically binds activated β1-integrins, to explore the regulation of integrin activation by 4N1K. We found that 4N1K not only induced binding of this antibody, but also of non-specific IgG control antibodies, to cell surfaces of both parental Jurkat and β1-integrin-deficient cells (Fig. 2). Our results indicate that 4N1K promotes the binding of a variety of antibodies to cell surfaces in a non-selective manner. In line with these observations, previous studies have shown that basic peptides had a propensity to bind a variety of IgG antibodies in a non-specific manner, especially when surrounded by aromatic residues [38]. However, because 4NGG did not mediate the same nonspecific interactions as 4N1K in our studies, the presence of two valine residues in 4N1K may increase the non-specific adhesive nature of this basic peptide. Consequently, this phenomenon alone disqualifies any meaningful interpretation of the effect of 4N1K on integrin activation in our assays, since all antibodies tested bound to the cells at levels exceeding even those of Mn^2+^-stimulated cells.

Previous reports of integrin activation using LIBS-type antibodies have obtained results that were remarkably similar to ours. Using the αIIbβ3-integrin LIBS antibody PAC-1, Chung *et al.* reported that 4N1K is a potent activator of platelet αIIbβ3 integrins [21]. The extent of PAC-1 binding mediated by 4N1K exceeded even that of epinephrine- or thrombin receptor peptide-induced effects [21]. This result is analogous to what we observed when comparing HUTS-21 binding to cells treated with 4N1K or with Mn^2+^ to induce integrin activation (Fig. 2). Initially, we had derived a similar erroneous interpretation that 4N1K was a potent activator of β1-integrins. It wasn't until our assays were repeated with a number of critical controls that the artifact became evident: 4N1K also induced high levels of HUTS-21 antibody binding in a β1-deficient cell line, and that of a generic IgG control antibody. It is worth noting that Chung *et al.* reported that the variant 4N7G peptide (KRFYVVMGKK) exhibited partial activation of integrins. Thus it is plausible that the charges on the amino acids following the VVM motif (glycine in 4N7G, the aromatic tryptophan in 4N1K) may play a role in the hyper-adhesive nature of 4N1K [21].

Fujimoto *et al.* also used PAC-1 to assess integrin activation and found that 4N1K increased platelet activation in a manner that did not require energy, indicating that the effects of 4N1K on antibody binding were passive and did not require cell signaling [33]. In a somewhat similar assay, Brittain *et al.* assessed the binding state of integrins using a combination of soluble fibronectin, followed by detection with an anti-fibronectin antibody, and found that 4N1K incubation increased antibody binding, which was taken as an indication of induced integrin activation [20]. However, in light of our findings, it is conceivable that the antibodies used in these assays did not bind to the intended specific targets, but rather non-specifically to 4N1K-treated cells, as was the case in our assays (Fig. 2 and 4). Indeed, we found that a variety of non-specific antibodies were able to bind to immobilized 4N1K (Fig. 3, and data not shown), further supporting our alternate interpretation of the integrin activation data using LIBS or any other antibody-based assays. We rationalize that since immobilized 4N1K can both engage cells or IgG antibodies, the pseudo integrin activation assays may have resulted from the binding of antibodies to 4N1K-coated cells.

We also investigated another property of 4N1K-treated cells which was shown to promote, or inhibit, cell adhesion to an integrin substrate such as fibronectin [19], [20]. Our adhesion assays determined that higher concentrations of 4N1K blocked cell adhesion to integrin ligands (Fig. 5 and 6), and reproduced the results of the majority of studies [10], [17], [18]. Surprisingly, our assays revealed that 4N1K-mediated inhibition of adhesion was not dependent on CD47; the adhesion of both CD47-expressing and non-expressing T-cells to fibronectin was blocked to a similar extent following incubation of cells with 4N1K (Fig. 6). Furthermore, by titrating the concentration of peptides incubated with cells, we found that lower concentrations of 4N1K actually stimulated cell adhesion to fibronectin (Fig. 6B). Following these unexpected results, we re-assessed previous published reports of cell adhesion experiments involving 4N1K and found that 4N1K blockade of adhesion to a variety of substrates occurred in cases where the peptide was used at 50 µM concentrations or higher [10], [15]–[18]. However, experiments that were performed at lower concentrations (5–10 µM) of the related 4N1 peptide were also found to increase cell adhesion to some of these same ligands [19]. Cell incubation with 4N1K-related peptides has also been shown to promote cellular aggregate formation that was differentially affected by peptide concentration; 25 µM 4N1 peptide resulted in a higher degree of cell aggregation when compared to 100 µM [22]. Exceptions to these observations include experiments with sickle red blood cells and platelets where higher concentrations of 4N1K stimulated cell adhesion [14], [20], [39], [40]. It should be noted that Brittain *et al*. found CD47 on sickle red blood cells to be conformationally different from those on normal reticulocytes, suggesting that some of these results may be cell-type dependent [41]. In the same study, the authors also found that 4N1K had opposite effects on sickle red blood cell adhesion under shear stress or static conditions [14].

Our assays also revealed that JinB8 cells were as efficient as JinB8-CD47 and Jurkat cells in binding to substrate-immobilized 4N1K (Fig. 4). The CD47-independent effects of 4N1K have also been observed in previous studies which found that Jurkat and JinB8 cells displayed no differences in 4N1K-mediated aggregation or adhesion [19], [22]. It should be noted that some studies have reported differences in responses to 4N1K between CD47^+/+^ and CD47^−/−^ cells or by using CD47-blocking antibodies [33], [42] which we cannot explain and may be attributed to specific differences in the assays.

Finally, the 4N1K epitope was shown to be buried within a hydrophobic region of the globular domain of TSP, leading to suggestions that it may be inaccessible for protein-protein interactions [28], [43]. Floquet *et al.* proposed that proximity between the TSP VVM motif and CD47 may unfold the TSP globular domain and provide a cleft for the IgV domain of CD47 to make contact [43], but this hypothesis has yet to be verified. Furthermore, plasmon resonance studies failed to detect an interaction between CD47 and the TSP signature domain which contains 4N1K's VVM motif in its native conformation [27]. Thus the collective evidence suggests that this globular domain of TSP may remain cryptic and does not unfold under normal physiological conditions. Further studies will be required to unravel the exact nature of 4N1K-mediated protein-protein interactions.

Given the evidence presented in this paper, we propose that the effects of 4N1K seen in multiple studies may not necessarily be the result of its interaction with CD47 as a ligand, but rather as a consequence of the hyper-adhesive nature of 4N1K. Others have cautioned against the use of 4N1K to study CD47-mediated effects [8], [44] and our study supports this opinion with carefully controlled assays. It should be noted that we do not challenge the role of CD47 in cell adhesion, spreading, or other functions shown to be mediated by it. Rather, we suggest that due to its non-specific effects, 4N1K is not an appropriate peptide ligand for use to study the effects of CD47 ligation by TSP, as its use can result in erroneous conclusions. Perhaps using a larger fragment of the TSP C-terminal binding domain would aid in eliminating the unwanted effects observed when using 4N1K, and would thus serve as a better peptide ligand to study CD47-mediated functions when and if the CD47-specificity can be affirmed.
